# Building a body of knowledge: sickle cell and libraries

**DOI:** 10.5195/jmla.2018.364

**Published:** 2018-01-02

**Authors:** Richard H. Nollan

Lemuel Whitley Diggs’s work reminds us how much of what we do is anchored in the work of our predecessors. Diggs had many heroes in his early education, among them Sir William Osler, physician-in-chief (1888–1905) at the newly formed Johns Hopkins Hospital and one of the founders of the Medical Library Association (MLA). Although the two never met, Diggs trained at the Johns Hopkins University School of Medicine in a program that was thoroughly informed by Osler’s clinical philosophy and medical expertise. Every physician carried the store of knowledge first acquired in medical school and then expanded by clinical practice.

The library for Osler was the storehouse of knowledge from science and the humanities, and he considered it an essential extension of every physician’s personal store, drawn upon as needed and expanded as new knowledge was gained. Diggs revered Osler and the professors at Hopkins, and carried this idea of the library with him throughout his career. Diggs wanted to be the best at something, and Osler’s teachings gave his innate ambition a focus in medicine that later informed his life. He graduated in 1926 and completed a two-year pathology residency at the University of Rochester in 1928 before moving to Memphis, Tennessee, to begin his career.

Diggs committed himself to studying sickle cell almost as soon as he arrived on the wards of the Memphis City Hospital in 1929. He was surprised to find that he could easily identify a significant number of patients with the disease, a much higher number than he had been taught in medical school, where it had still been considered a rare disorder. He could only wonder if he had either discovered an unexplained concentration in Memphis or if sickle cell was much more common than previously thought. The reason for this, he wrote, was because “the condition is unrecognized, and a knowledge of the blood picture is necessary for its recognition” [1].

The disorder was not rare because the incidence was so low, but rather because a greater effort was required by physicians to understand what to look for. In the 1920s, sickle cell could only be diagnosed on the basis of a smear viewed through a microscope. However, few physicians in Memphis (or elsewhere) were comfortable using a microscope or a clinical laboratory for patient care. With the poor state of knowledge of sickle cell at this time, the disease could masquerade as many other non-hematological illnesses, which led Diggs to publish both as a way to communicate new knowledge and to inform others of the importance of putting sickle cell on the differential diagnosis list. The urge to know everything when beginning a new project extended to his research as it did to many contemporaries, and like them, he wanted to be able to add to and expand on what they did before him. Once he saw sickle cell in a new light, his reaction was to set about creating a clear definition of the disease and to establish the prevalence of the disease in the general population.

New diseases and therapies were being discovered, and he believed deeply in the power of science to improve knowledge and human health. On social issues, he was a moderate but believed that with time, science could transform social problems, too. Diggs had participated in George Whipple’s dog trials at the University of Rochester that led to the discovery of the beneficial effects of liver extract in ameliorating and reversing the symptoms of pernicious anemia. Years later, it became understood that the disorder was caused by the malabsorption of vitamin B12, but his observation of the dramatic recovery by patients from this debilitating and fatal illness deeply impressed him [2].

Diggs always relied on the literature to guide his work, and he would use it to help him understand the clinical, laboratory, anatomical, and pathological manifestations of sickle cell disease. This material was an indispensable part of his working life. He would routinely refer in his publications to the number of journal articles available on its subject and would include either a complete list of those articles or list the significant ones. He regularly worked in the library using the interlibrary loan service to get articles and the *Cumulative Index Medicus* to identify articles that he needed. There were few articles on sickle cell in 1929, but he acquired all of them and afterward continued to keep all the annotated articles that he could find in a file cabinet so that anyone who needed to could find what they needed.

His training at the Johns Hopkins School of Medicine taught him to read everything available on a research subject, and in this, he used the medical library and its resources to obtain everything he could find on sickle cell, a habit he would pursue until the 1970s. He and members of his staff often annotated the articles with marginal comments. In one instance, an author made the assertion that “The true sickle cells in the blood of an active case are absolutely unaffected by oxygen.” To this, Diggs wrote a firm “NO!” in the margin [3]. His purpose was not only the practical one of showing where he had obtained his information, but also to teach the most effective approach to any clinical research problem, namely to begin with what was already known about a particular problem.

Believing that scientific information should be freely available, Diggs used local help to copy and bind into sixty-four volumes all the articles that he could find on sickle cell from 1910, when the disorder was first described, to 1970. Sets of these articles were donated to the University of Tennessee Health Sciences Library, St. Jude Children’s Research Hospital, and the National Library of Medicine [4]. Today, of course, such copying and distribution raises serious questions of copyright infringement, but in that print-only world, there was so little concern that the question about copyright more than likely never occurred to anyone.

**Figure 1:**
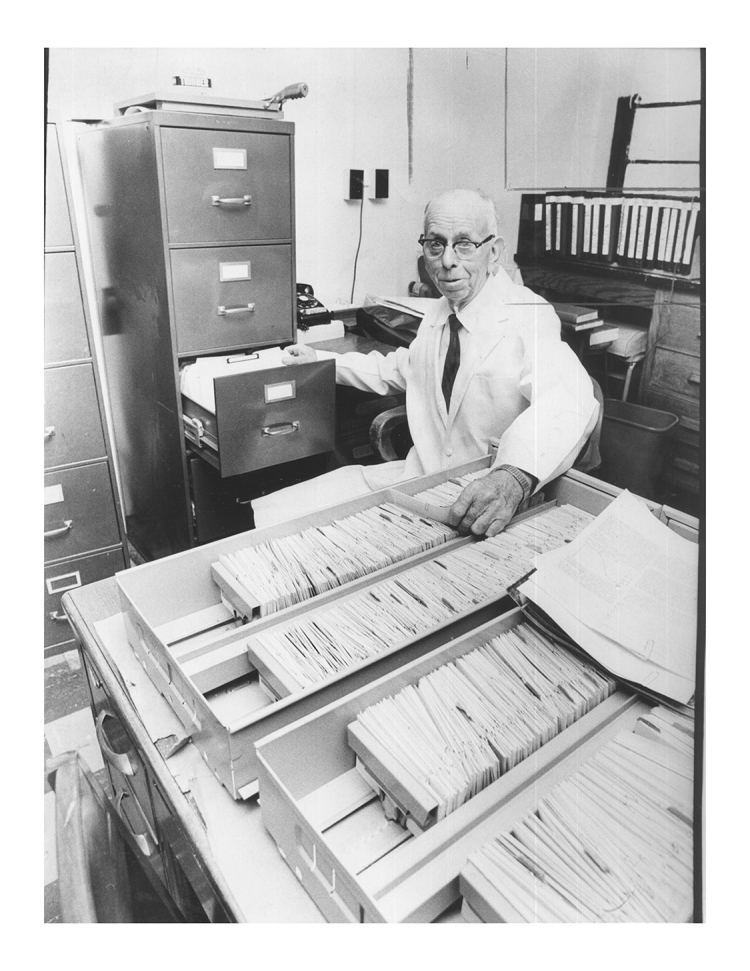
Lemuel Whitley Diggs with his sickle cell database

In the 1950s, Diggs started the first Sickle Cell Center in the country in a room provided by the university with what equipment he could gather together. But its purpose was to focus the research on finding a cure for sickle cell. True to his training, he was not only interested in the physiological aspects of the disease, but also in the patient’s ability to learn in school, to hold a job, and to continue in their home life. To this end, he created a database on four-by-six-inch index cards of everyone in the Memphis region, including Tennessee, Arkansas, and Mississippi (Figure 1). The thousands of cards created for this project were assembled over many years and with much attention to detail, and it would be unwieldy except for an army of assistants, much less one physician with staff support.

Today, we would see this as something only a computer could organize, but Diggs persisted in finding the answers to this disease. The picture of him is telling, with one hand on the patient data that he had collected and the other in a file cabinet containing copies of the medical literature that he had acquired from the library. As a tribute to his endeavors and those who worked for him, the center was renamed the Diggs-Kraus Sickle Cell Center in 1994 as the oldest center of its kind in the country. Diggs’s career is likewise a testimony to the role of libraries throughout health care education and practice.
